# Noninvasive 40-Hz Light Flicker Rescues Circadian Behavior and Abnormal Lipid Metabolism Induced by Acute Ethanol Exposure *via* Improving SIRT1 and the Circadian Clock in the Liver-Brain Axis

**DOI:** 10.3389/fphar.2020.00355

**Published:** 2020-03-25

**Authors:** Youli Yao, Wenjiang Zhang, Ruibo Ming, Qiyu Deng, Along Zuo, Shengli Zhang, Ying Ying, Yingying Zhao, Junxian Ma

**Affiliations:** ^1^Department of Physiology, School of Basic Medical Sciences, Shenzhen University Health Sciences Center, Shenzhen University, Shenzhen, China; ^2^School of Information Engineering, Shenzhen University, Shenzhen, China; ^3^Key Laboratory for Natural Resource of Changbai Mountain and Functional Molecules, Ministry of Education, Yanbian University, Yanji, China

**Keywords:** 40-Hz light flicker, sirtuin 1, circadian clock, alcoholic liver steatosis, suprachiasmatic nucleus

## Abstract

Sirtuin 1 (SIRT1) is a protein deacetylase with important cellular functions, as it regulates numerous processes, including the circadian rhythm in peripheral tissues. Efforts are ongoing to reveal how Sirt1 can be used to treat diseases, such as alcoholic liver disease (ALD), Alzheimer's disease, and liver fibrosis. We have recently shown that noninvasive exposure to 40-Hz light flicker activates hypothalamic SIRT1 gene expression, thereby regulating the central circadian clock. This study investigated the effects of 40-Hz light flicker in a mouse model of ALD. RNA sequencing (RNA-seq) analysis was performed to explore the potential pathways affected by 40-Hz light flicker. We found that 40-Hz light flicker significantly decreased the acute ethanol-induced increases in serum alanine aminotransferase (ALT) and serum triglyceride (TG) levels and reduced fat-droplet accumulation in mouse livers. Additionally, 40-Hz light flicker significantly suppressed ethanol-induced increases in sterol regulatory element binding protein 1 (SREBP-1) and fatty acid synthase (Fasn) levels. Furthermore, the ethanol induced significant decreases in both Sirt1 levels and phosphorylation of adenosine monophosphate-activated protein kinase subunit (AMPKα), compared with those in the control group. Strikingly, pretreatment with 40-Hz light flicker ameliorated such ethanol-induced decreases in SIRT1 levels and AMPKα phosphorylation. In addition, ethanol-induced increases in levels of brain and muscle arnt-like protein-1 (BMAL1), circadian locomotor output cycles kaput (CLOCK), and period 2 (PER2) were reversed by 40-Hz light flicker. RNA-seq analysis revealed significant differences in expression of genes related to the AMPK signalling. Moreover, ethanol consumption altered mRNA levels of Sirt1 and circadian genes in the suprachiasmatic nucleus (SCN), indicating that ethanol influenced central pacemaker genes; however, 40-Hz light flicker reversed these ethanol-induced changes. Taken together, our findings demonstrate that 40-Hz light flicker rapidly influence the SCN and exhibits inhibitory properties on hepatic lipogenesis, indicating that 40-Hz light flicker has therapeutic potential for preventing alcoholic liver steatosis.

## Introduction

The number of patients with chronic liver disease has increased rapidly in recent years (Llorente et al., 2017). Chronic liver diseases, such as cirrhosis and liver cancer, are among the top causes of deaths worldwide (Kim et al., 2014). Approximately 50% of all liver-related deaths are related to alcohol (Rehm et al., 2013). Acute alcoholic liver disease (ALD) is caused by excessive intake of alcohol (Gustot and Jalan, 2019). Ethanol consumption disrupts lipid metabolism, leading to excessive accumulation of triglycerides in the liver, which aggravates damage to hepatocytes (Wang et al., 2016b). If drinking continues, acute alcoholic fatty liver will develop to hepatic fibrosis, cirrhosis, or even hepatic cancer. Unfortunately, there have been no effective treatments for ALD other than alcohol withdrawal. Therefore, discovery and development of safe and effective therapies are urgently needed.

Sirtuin 1 (SIRT1) is a histone deacetylase that modulates the activity of a number of transcription factors (Rahman and Islam, 2011). In cellular metabolism, SIRT1 couples with circadian machinery to modulate cellular activity and stability. Brain and muscle arnt-like protein-1 (BMAL1) is an important component of the circadian clock. Ethanol-diet-fed mice with liver-specific knockout of BMAL1 develop more severe liver steatosis and injury, as well as a simultaneous suppression of both *de novo* lipogenesis and fatty acid oxidation (Zhang et al., 2018a). The activation of BMAL1 regulates cyclic variation in levels of various metabolic genes that drive the metabolism of lipid, glucose, and cholesterol (Asher and Sassone-Corsi, 2015). SIRT1 promotes the deacetylation and degradation of period 2 (PER2) by binding to BMAL1: circadian locomotor output cycles kaput (CLOCK) heterodimers (Asher et al., 2008). PER2 is a member of the period family of genes and regulates daily rhythms of metabolism, locomotor activity, and other behaviors. The degradation of PER proteins prevents the formation of large protein complexes, thereby inhibiting the transcriptional activity of the BMAL1: CLOCK heterodimer (Nakahata et al., 2008).

SIRT1 relies on nicotinamide adenine dinucleotide (NAD^+^) histone deacetylase to regulate energy metabolism, stress responses, aging, and maintenance of genomic integrity. Adenosine monophosphate-activated protein kinase (AMPK) is a metabolic sensor that regulates cellular energy homeostasis and interacts with SIRT1. The overexpression of SIRT1 causes serine/threonine kinase 11 (also known as liver kinase B1, LKB1) translocation from the nucleus to the cytoplasm, resulting in LKB1 activation (Lan et al., 2008). LKB1 is constitutively active and functions as an upstream kinase for Thr172 of AMPK (Alexander and Walker, 2011). Activated LKB1 then activates AMPK through phosphorylation. AMPK activates SIRT1 by transcription of nicotinamide phosphoribosyl transferase. SIRT1 inhibits transcription factor activity, thereby regulating lipid-metabolism signaling pathways. Sterol regulatory element binding protein 1 (SREBP-1) is a master transcription factor that regulates lipogenic enzymatic expression, including fatty acid synthase, acetyl-CoA carboxylase (ACC), and stearoyl-CoA desaturase-1 (Purohit et al., 2009) (You et al., 2002). Peroxisome proliferator-activated receptor-α (PPARα) is a key transcriptional regulator of lipolytic enzymes and uncoupling proteins (Mandard et al., 2004). Phosphorylation of AMPK inhibits lipogenesis and accelerates fatty acid oxidation by modulating the activities of SREBP1 and PPARα. Therefore, this study postulated that SIRT1/AMPK and BMAL1 may represent key therapeutic targets for reducing disease progression.

Cyclic variation in molecular rhythms are self-sustaining and self-generating (Nagoshi et al., 2004). However, mammalian central peripheral clocks respond to light/dark changes through diverse pathways. Among the mechanisms involved are photic inputs transduced by retinal neurons. These inputs are transmitted to the suprachiasmatic nucleus (SCN) through the retinohypothalamic tract (Takahashi, 2017). The SCN cells process these inputs and convey this information to neurocircuitry that ensure peripheral regulation of metabolic activity (Panda, 2016). Light innervation alters behavioral and metabolic outputs—including feeding patterns, energy expenditure, and corticosterone secretion—*via* changes in the expression of clock genes within ventromedial hypothalamic (VMH) neurons, as well as within liver and brown tissues (del Río-Martín et al., 2019).

Long-term clinical observations have demonstrated that bright light therapy can significantly improve mood, sleep, and daily activities in patients with Alzheimer's disease, and its application is noninvasive, affordable, and convenient (Perusini et al., 2017) (Iaccarino et al., 2016). Tsai et al. found that light therapy at a frequency of 40 Hz can inhibit the production of amyloid beta (Aβ) and activate microglia to accelerate the removal of existing Aβ (Iaccarino et al., 2016). Other studies have found that light can restore memory in a model of Alzheimer's disease. However, little is known about the efficacy of 40-Hz light flicker on circadian parameters, and it remains unclear whether 40-Hz light flicker protects the liver from acute alcoholic hepatic injury. Hence, the aim of this study was to investigate the hepatoprotective effects of 40-Hz light flicker on the regulation of lipid accumulation and circadian clocks *via* SIRT1/AMPK and BMAL1 in mice with acute alcohol exposure.

## Materials and Methods

### Animals

Ten-week-old male C57BL/6 mice (22–25 g) were obtained from the Guangdong Medical Laboratory Animal Center (Guangzhou, China) (SPF, SCXK (Yue) 2018-0002, Guangzhou, China). The experiment procedures were approved by the Institutional Animal Care and Use Committee of Shenzhen University (Resolution number, 2019005). All efforts were made to reduce animal suffering. Mice were randomly divided into the following four groups (n = 10 per group): a control group, ethanol group (ETOH), 40-Hz light flicker group (40 Hz) and an ETOH plus 40-Hz light flicker group (ETOH + 40Hz). In this study, a light source device was fabricated using the following spectrum (**Figure S1A**). The four sides of the exposure boxes were wrapped with black cloth or plastic as shown in **Figure S1B**. This strategy blocked unwanted light from other sources and, hence, ensured that light was only from the LED. The LED source was placed above the box. Mice were acclimatized to the lab for 1 h. Thereafter, ETOH mice were subjected to ETOH (5 g/kg body weight) three times within 24 h, whereas control mice and 40-Hz group mice were administrated (by gavage) with an equal volume of saline. The 40-Hz group and ETOH + 40-Hz group were exposed to 40-Hz light flicker for 1 h (**Figure S1C**) at 8:00 (defined as zeitgeber time zero [ZT0]) from ZT0 to ZT1 two times within 24 h. During stimulation, mice were allowed to freely explore or rest around the exposure box. The mice were euthanized at 4 h after the last ethanol dosing. The brain, blood, and liver tissue were collected from each euthanized mouse. All animal experiments and maintenance were approved by the Laboratory Animal Ethics Committee of Shenzhen University.

Ten-week-old male C57BL/6 mice (n = 4) were used for recording. Ethovision XT is the applied video tracking software and used to record and analyze locomotor behavior. Spontaneous locomotor activity was defined as the moving distance per unit time (3 min). Mice were videotaped for 4 days as basal measurements. For the following 24 h, all mice were given ETOH (5 g/kg body weight), and they were divided into “ETOH groups” and “ETOH + 40-Hz groups” based on whether a 40-Hz light flicker treatment was given. The mice were continuously recorded for 5 days.

### Antibodies and Reagents

Anti-SREBP1 (ab28481), anti-GAPDH (ab8245), anti-CLOCK (ab3517), anti-BMAL1 (ab228594), and anti-SIRT1 (ab110304) were purchased from Abcam (Cambridge, MA, USA). Antibodies against AMPKα (cs5831) and p-AMPKα (cs2535) were purchased from Cell Signaling Technology (Beverly, MA, USA). Antibodies against PER2 (AB2202) was purchased from Millipore (Billerica, MA, USA). Horseradish peroxidase (HRP)-conjugated goat anti-rabbit and goat anti-mouse antibodies were purchased from Abcam.

### Serum Aminotransferase and Triglyceride Measurements

Blood samples were separated by centrifugation at 3,000 rpm for 30 min and levels of alanine aminotransferase (ALT) and triglycerides (TGs) in blood sera were measured using an Automatic Chemistry analyzer (SPOTCHEM, sp-235 4430, Japan).

### Immunofluorescent Staining

Immunofluorescent staining for p-AMPKα (cs2535), SIRT1 (ab110304), SREBP1 (ab28481), CLOCK (ab3517), BMAL1 (ab228594), and PER2 (AB2202) were performed on frozen sections. Frozen sections were fixed in acetone/methanol (1:1), dried, dehydrated, and blocked with normal goat serum for 1 h to reduce nonspecific binding. The tissue sections were incubated with primary antibodies (1:200) overnight at 4°C. Subsequently, the slides were incubated with secondary antibodies (1:200) for 1 h. After counterstaining with DAPI, fluorescence was viewed and imaged *via* ultra-high resolution confocal microscopy (LSM880, CARL ZEISS, Germany).

### RNA-Sequencing Analysis

Isolated RNA was subsequently used for RNA-seq analysis. cDNA library construction and sequencing were performed by the BGI-Shenzhen using the BGISEQ-500 platform. We identified differentially expressed genes (DEGs) between samples and performed clustering analysis and functional annotation. Genes with ≥ twofold change and a false discovery rate (FDR) of ≤ 0.001 were considered to be statistically significant.

### Western Blot Analysis

The liver was lysed by RIPA buffer (Sigma) with the presence of protease and phosphatase inhibitors. The supernatants were collected and protein concentrations were measured by BCA Protein Assay Kit (BIO RAD). Equal amounts of protein were separated by SDS/PAGE and then transferred to PVDF membranes (GE healthcare, Freibury, Germany). The membranes were incubated with specific primary antibodies, at 4°C overnight. HRP-conjugated secondary antibodies (1:8,000) were from Abcam. Immunoreactive bands were revealed by enhanced chemiluminescence (Clarity Western ECL Subs kits, BIO RAD) and then stripped and reprobed with anti-GAPDH for the loading control. Band intensities were quantified by densitometry using Quantity One software (BIO RAD).

### Quantitative Real-Time PCR

The extraction of total RNA from hepatic tissue, reverse transcription of total RNA was performed using commercial reagent kit (TaKaRa, Dalian, China). The relative gene expression was determined by real-time PCR with Power SYBR^®^ Green PCR Master Mix (Bio-Rad, Hercules, CA, USA) on an Analytik Jena QPCR System. The relative expression of genes was analyzed by method of 2^-ΔΔCt^. The specific primers used for the gene expression analysis are shown in **Table 1**.

**Table 1:** Primer sequences used for quantitative real-time PCR analysis.

Genes	Forward (5'-3')	Reverse (5'-3')	
mSrebp1	5'-CTTAGCCTCTACACCAACTG-3'	5'-AGGAATACCCTCCTCATAGC-3'
mFasn	5'-ATTGTGGATGGAGGTATCAAC-3'	5'-CTGGTAGGCATTCTGTAGTG-3'
mClock	5'-TCACCACGTTCACTCAGGACA-3'	5'-AAGGATTCCCATGGAGCAA-3'
mBmal1	5'-ACAATGAGCCAGACAACG-3'	5'-TTCCCATCTATTGCGTGT-3'
mPer2	5'-CACTTGCCTCCGAAATAA-3'	5'-ACTACTGCCTCTGGACTGG-3'
mSirt1	5'-TGATTGGCACCGATCCTCG-3'	5'-CCACAGCGTCATATCATCCAG-3
mGAPDH	5'-CTTGTGCAGTGCCAGCC-3'	5'-GCCCAATACGGCCAAATCC-3'

### Statistical Analyses

All data were expressed as the mean ± SD. Statistical significance was analyzed by one-way ANOVA followed by a Student's *t* test. Data were considered significant when P < 0.05. Calculations were performed using GraphPad Prism (GraphPad Software, San Diego, CA, USA).

## Results

### 40-Hz Light Flicker Ameliorates Ethanol-Induced Liver Injury

**Figure 1:**
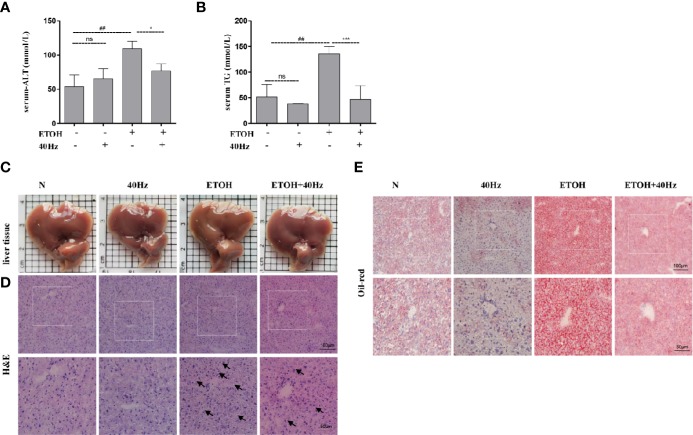
Forty-hertz light flicker reduces the features of ethanol-induced liver injury. Serum alanine aminotransferase (ALT) **(A)** and triglyceride (TG) **(B)** contents. Each value is expressed as the mean ± SD (n = 6 in each group). Liver appearance pictures **(C)**, Hematoxylin and Eosin (H&E) **(D)** and Oil Red O staining **(E)** were performed with samples obtained at 4 h after the last ethanol administration (200 × original magnification). ^##^*P* < 0.01 significantly different from normal group; ^*^P < 0.05, ^***^P < 0.001, significantly different from ethanol alone group; one-way ANOVA followed by Tukey's test. All histograms represent the mean ± SD of five independent assays. NS, nonsignificant.

Serum ALT and TG levels were used to evaluate the effects of ethanol and/or 40-Hz light flicker in the liver. As shown in **Figures 1A, B** ethanol significantly increased serum ALT and TG, which were reversed by 40-Hz light flicker. Structurally, the liver tissues of control mice were reddish-brown, and their surfaces were soft, smooth, elastic, and not easily broken (**Figure 1C**). However, the liver tissues in ethanol-treated mice were yellowish, greasy, easily broken, and weak in elasticity. With 40-Hz light flicker treatment, the livers of ethanol-treated mice were reddish brown, normal in color, and elastic. Application of 40-Hz light flicker alone did not induce any significant changes compared with parameters in the control group.

Next, hematoxylin and eosin (H&E) staining was performed to assess the extent of histopathological injury (**Figure 1D**). Acute ethanol exposure resulted in massive fat vacuoles compared with those of the control group. These changes were significantly reversed by 40-Hz light flicker pretreatment. To further evaluate hepatic steatosis, liver sections were subjected to Oil-Red-O staining. Substantial fat droplets formed in the ethanol group but exposure to 40-Hz light flicker antagonized such ethanol-induced hepatic steatosis (**Figure 1E**). Unexpectedly, the lipid droplets of alone 40-Hz pretreated mouse livers were much smaller and less abundant (**Figure 1E**). These results reveal that 40-Hz light flicker suppressed the effects of ethanol on hepatic steatosis and lipid metabolism.

### Effects of 40-Hz Light Flicker and Acute Ethanol on SIRT1 Signaling

**Figure 2:**
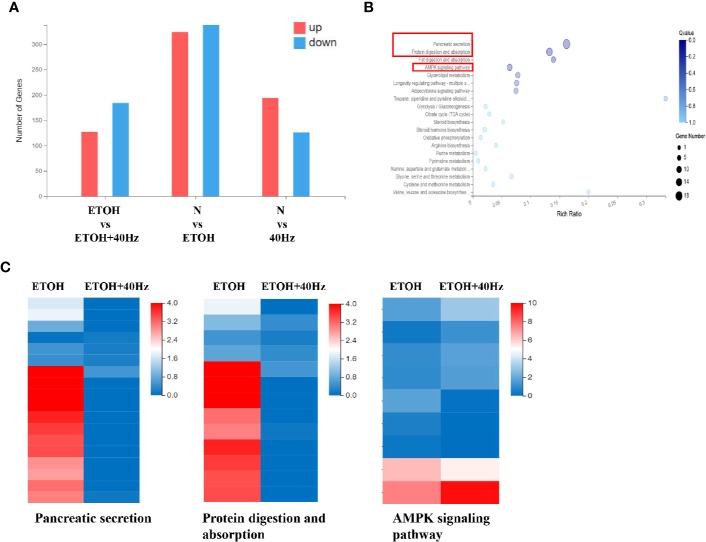
Effects of 40-Hz light flicker and acute ethanol challenge on gene expression. **(A)** Differentially expressed genes of livers treated with ethanol and/or 40-Hz light flicker, as indicated by RNA-seq analysis (n = 3). **(B)** Gene ontology (GO) analysis of target genes, showing biological processes affected by 40-Hz light flicker treatment (n = 3). The *x*-axis is the enrichment ratio (Rich Ratio = Term Candidate Gene Num/Term Gene Num). The *y*-axis represents the GO terms. The size of each bubble indicates the number differentially expressed genes annotated to a GO Term. The color represents the enriched Q value. The darker the color, the smaller the Q value. **(C)** Heatmap of genes upregulated or downregulated from 40-Hz light flicker and/or ethanol treatment in liver tissue (n = 3). The horizontal axis is the log2 (expression value +1) of the sample, and the vertical axis represents the corresponding gene. Patches that have a redder color denote higher expression, whereas patches with bluer colors denote lower expression.

After receiving various treatments, the mice were euthanized and their livers were harvested. We then compared liver RNA levels from the ETOH group with those of the control, 40-Hz, and ETOH + 40-Hz groups (**Figure 2A**). RNA sequencing (RNA-seq) analysis showed significant changes in 311 mRNA levels in the ethanol group compared with the ETOH + 40-Hz group **(Figure 2A)**. Kyoto Encyclopedia of Genes and Genomes (KEGG) pathway analysis identified 19 significant signaling pathways, including those involved in fat absorption/digestion, protein absorption/digestion, pancreatic secretion, and AMPK signaling **(Figures 2B, C)**.

**Figure 3:**
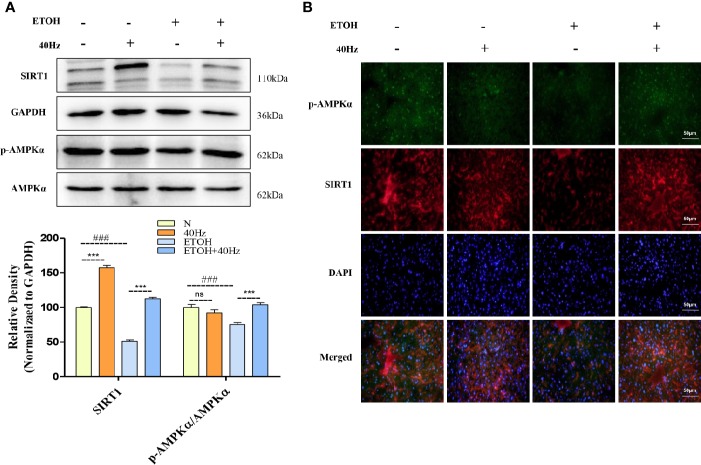
Effects of 40-Hz light flicker and acute ethanol challenge on the Sirtuin 1(SIRT1)–adenosine monophosphate-activated protein kinase subunit (AMPKα) signalling. **(A)** Protein expression levels of SIRT1, AMPKα, and phospho-AMPKα of mice liver were determined at 4 h after the last ethanol administration by five independent Western blots. **(B)** SIRT1 (red), phospho-AMPKα staining (green) and nuclei with DAPI (blue) are shown (400× original magnification, n = 6). ^###^*P* < 0.001 significantly different from normal group; ^***^P < 0.001, significantly different from ethanol alone group; one-way ANOVA followed by Tukey's test.

The development of fatty liver following ethanol uptake is regulated by lipid homeostasis, a process in which SIRT1 plays a role. **Figure 3** shows that the protein expression of SIRT1 was decreased following ethanol exposure. Furthermore, 40-Hz light flicker increased SIRT1 protein and mRNA levels, especially from single 40-Hz light flicker administration, indicating that 40-Hz light flicker may act as a SIRT1 activator (**Figure 3** and **Figure S3**). AMPK participates in glucose and lipid metabolism by interacting with SIRT1. We found that ethanol exposure markedly decreased p-AMPKα levels in the liver compared with those in the control group, whereas 40-Hz light flicker promoted p-AMPKα expression relative to that in ETOH group. Similar to the Western blot results, decreases of SIRT1 and p-AMPKα levels caused by acute alcohol-induced liver injury were prevented by 40-Hz light flicker, as examined by immunofluorescence (**Figure 3B**).

### 40-Hz Light Flicker Prevents Ethanol-Induced Increases in SREBP-1

**Figure 4:**
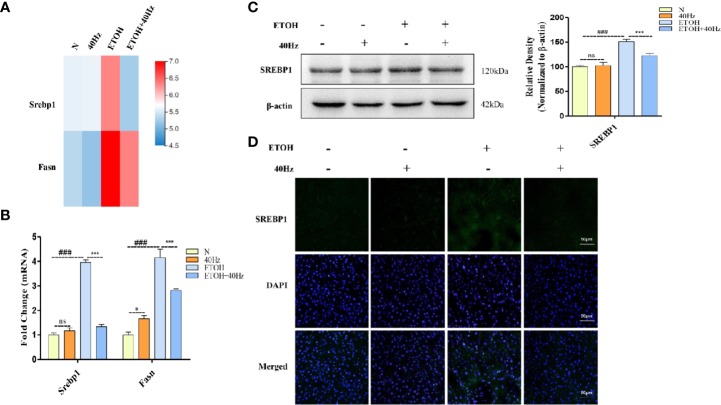
Forty-hertz light flicker suppressed sterol regulatory element binding protein 1 (SREBP-1) activations induced by acute ethanol challenge. **(A)** Heatmap of srebp1 and fatty acid synthase (Fasn) with 40 Hz light-flickering or ethanol treatment of mice liver tissue (n = 3). **(B)** The mRNA expression of srebp1 and Fasn. **(C)** Protein expression of SREBP1 was determined by five independent Western blots. **(D)** SREBP1 staining (green) and nuclei with DAPI (blue) are shown. ^###^P < 0.001 significantly different from normal group; ^*^P < 0.005, ^***^P < 0.001, significantly different from ethanol alone group; one-way ANOVA followed by Tukey's test. NS, nonsignificant.

RNA-seq analysis revealed significant differences in Srebp1 and Fasn between mice that received 40-Hz light flicker treatment and those treated with ethanol (**Figure 4A**). It has been reported that SREBP-1 is a key driver of fatty acid synthesis. We, therefore investigated the impact of 40-Hz light flicker on SREBP-1. Alcohol promoted the expression of SERBP-1 at both mRNA and protein levels (**Figures 4B, C**). In contrast, 40-Hz light flicker significantly reversed these effects. However, treatment with only 40-Hz light flicker had no effect on SREBP-1. In addition, liver histological sections were analyzed by immunofluorescence to assess the distribution of SREBP-1 in the liver. SREBP-1 showed positive staining in liver sections from the ethanol group (**Figure 4D**), whereas the staining was weakened by 40-Hz light-flicker treatment (**Figure 4D**). FASN plays an important role in lipogenesis by synthesizing saturated long-chain fatty acids. Therefore, we assessed whether 40-Hz light-flicker treatment was related to FASN inhibition. **Figure 4B** shows that ethanol challenge enhanced Fasn mRNA levels, but 40-Hz light flicker treatment decreased its expression of ethanol-treated mice. These results demonstrate that 40-Hz light flicker blocked lipogenesis by inhibiting SREBP-1 and FASN expression in the liver after ethanol exposure.

### 40-Hz Light Flicker Resets the Circadian Rhythm in Mice Receiving Acute Alcohol Exposure

**Figure 5:**
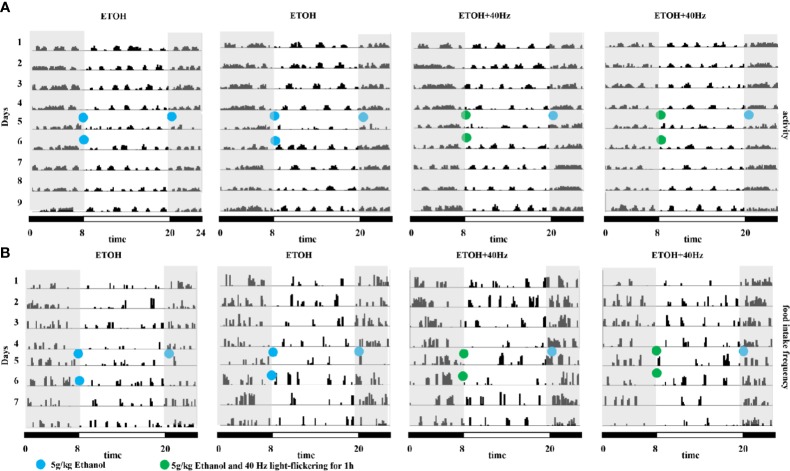
Effects of 40-Hz light flicker on average locomotor activity and food intake in mice provided with acute alcohol exposure. **(A)** Representative average locomotor activity records. Locomotor activity was defined as the moving distance per unit time (3 min) of each group. **(B)** Representative frequencies of food intake. Food-intake frequency was defined as the number of episodes of food intake per unit time (3 min) of each group. Blue circles represent gavage of 5 g/kg of alcohol; green circles represent gavage of 5 g/kg of alcohol and 40-Hz light flicker for 1 h. Double-plotted actograms are shown; black and white bars on the bottom indicate dark and light periods, respectively. Gray shading indicates dark periods.

In other experiments, we assessed patterns of spontaneous locomotor activity under control light-dark conditions (12-h light/12-h dark). Compared with that during the light period, wild-type mice were more active during the dark period, displaying occasional activity bouts during the light (**Figure 5A**); this result is consistent with the nocturnal nature of mice. Under control light-dark conditions, daily diurnal locomotion rhythms were stable. We further explored whether circadian clock patterns were altered in ethanol-treated mice. We found that ethanol-treated mice displayed activity bouts during the light and low activity during the dark (**Figure 5A**). Furthermore, 40-Hz light stimulation suppressed the activity of ethanol-treated mice during the light whereas ethanol exposure suppressed feeding frequency during the dark (**Figure 5B**). As expected, 40-Hz light stimulation increased feeding frequency of ethanol-treated mice. Despite the few striking differences compared with parameters in wild-type mice, the distribution of locomotor activity of ETOH + 40-Hz groups were similar throughout the 8-day recording period.

### 40-Hz Light Flicker Attenuates Ethanol-Induced Disruption of the Circadian Clock Pathway

**Figure 6:**
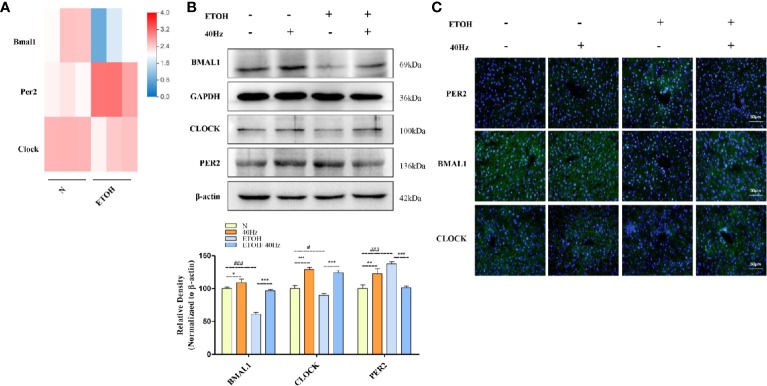
Forty-hertz light flicker attenuates dysregulation of the circadian clock pathway in alcohol exposure mice. **(A)** Heatmap of brain and muscle arnt-like protein-1 (Bmal1), period 2 (Per2), and circadian locomotor output cycles kaput (Clock) with ethanol treatment of mice liver tissue (n=3). **(B)** Protein expression levels of PER2, BMAL1, and CLOCK of mice liver were determined at 4 h after the last 40-Hz light flicker administration by five independent Western blots. **(C)** Expression of PER2, BMAL1, and CLOCK in mouse livers were evaluated by immunofluorescence staining (400× original magnification) (n = 6). ^#^*P* < 0.05, ^###^*P* < 0.001 significantly different from pair-fed group; ^*^P < 0.05, ^**^P < 0.01, ^***^P < 0.001, significantly different from ethanol group; one-way ANOVA followed by Tukey's test.

It is well documented that ethanol alters the circadian system in the liver (Zhang et al., 2018a). To explore the molecular mechanisms of this phenomenon, RNA-seq was performed to detect changes in mRNA levels in control and ETOH mice *via* a BGISEQ-500 platform (**Figure 6A**). The RNA-seq results provided information on the genes involved in the circadian pathway (**Figure 6A**). Bmal1 and Clock mRNA levels were decreased in the ethanol group (**Figure 6A**). To further evaluate the protein expression patterns of BMAL1 and CLOCK, we performed Western blotting and immunofluorescent analyses (**Figures 6B, C**). In the ethanol group, acute ethanol administration downregulated BMAL1 and CLOCK protein levels (**Figures 6B, C**). In contrast, 40-Hz light-licker treatment significantly increased protein expressions of BMAL1 and CLOCK in comparison with those in ethanol-group mice.

It has been demonstrated that SIRT1 interacts with CLOCK/BMAL1 to promote deacetylation and degradation of PER2 in the liver (Wang et al., 2016a). Therefore, we next measured changes in PER2 expression. Acute exposure to ethanol caused a marked upregulation of liver PER2 protein levels, as determined by Western blotting and immunofluorescent assays (**Figures 6B, C**). In contrast, 40-Hz light-flicker treatment downregulated PER2 levels in comparison with those in ethanol-treated mice. Taken together, these results show that 40-Hz light flicker is sufficient to correct circadian gene disruption in the liver from ethanol-induced hepatosteatosis.

### Effects of 40-Hz Light Flicker on mRNA Levels of Clock Genes in the SCN

**Figure 7:**
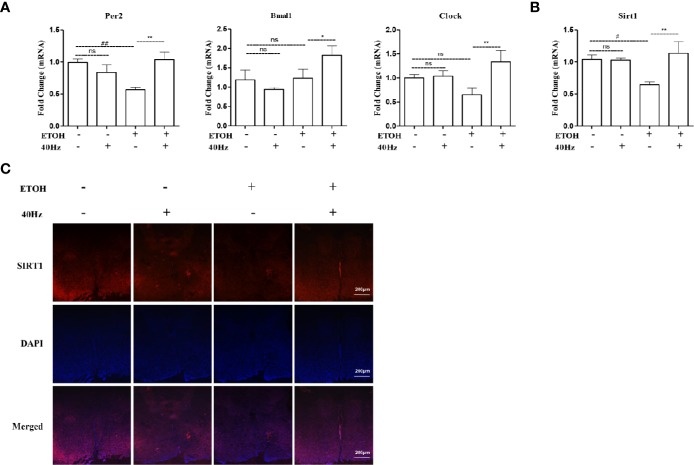
Effects of 40-Hz light flicker on clock genes in the suprachiasmatic nucleus (SCN). **(A)** The mRNA expression of Per2, Bmal1, and Clock (n = 6). **(B)** The mRNA expression of Sirt1 (n = 6). **(C)** SIRT1 (red) and nuclei with DAPI (blue) are shown (100 × original magnification). ^#^*P* < 0.05, ^##^*P* < 0.01 significantly different from normal group; ^*^P < 0.005, ^**^P < 0.01, significantly different from ethanol alone group; one-way ANOVA followed by Tukey's test. All histograms represent the mean ± SD of five independent assays. NS, nonsignificant.

To determine if ethanol-dependent alterations in the liver circadian clock were due to dysregulation of the central circadian clock in the SCN, we next examined mRNA levels of Bmal1, Clock, and Per2 in SCN samples. We observed reductions in the mRNA levels of key clock regulators, including Bmal1, Clock, and Per2 in ethanol-treated mice, whereas 40-Hz light flicker treatment activated the circadian pathway by elevating these mRNA levels (**Figure 7A**). In addition, the gene expression of Sirt1 was downregulated from ethanol exposure, where this ethanol-induced inhibitory effect was ameliorated *via* 40-Hz light flicker treatment (**Figure 7B**). Immunofluorescent analysis also demonstrated a positive regulation of 40-Hz light flicker on Sirt1 in the SCN (**Figure 7C**). These results suggested that 40-Hz light flicker activated Sirt1 and corrected ethanol-induced disruption of the central circadian rhythm in SCN, suggesting that 40-Hz light flicker may reset liver clock by first affecting the function of the central pacemaker.

## Discussion

ALD is comprised of a broad spectrum of disorders, ranging from steatosis, alcoholic hepatitis and fibrosis, and even to hepatocellular caner. Management of ALD is currently at a limited pharmacotherapy stage (Addolorato et al., 2009). The recommended drugs by the FDA for ALD include pentoxifylline, prednisolone, disulfiram, and naltrexone (M Edwards et al., 2011) (Vuittonet et al., 2014). Despite their modest success in controlling the symptoms of ALD, the survival rate and curative effects for severe ALD cases are low. Thus, first-line therapeutic interventions include nutritional optimization and abstinence from alcohol.

In this study, we show, for the first time, that 40-Hz light flicker activates SIRT1 in the SCN to regulate central circadian control in the mouse brain to reset the liver clock and lipid metabolism. We found that 40-Hz light flicker directly activated BMAL1 transcription by SIRT1 to regulate the magnitude of SCN gene expression of BMAL1 and other circadian regulatory proteins. Our findings on the liver-protective effect induced by alteration of SIRT1 are congruent with the reported effects of SIRT1 on liver function (by SIRT1 knockout or overexpression) (Timmers et al., 2011) (Purushotham et al., 2009). Thus, our results reveal that 40-Hz light flicker is a promising therapeutic intervention for linking changes in SIRT1 expression with modulation of the circadian clock.

The pathogenesis of alcoholic fatty liver begins with the development of hepatic steatosis (Zhang et al., 2018b). Alcohol exposure can increase fat production by directly or indirectly regulating SIRT1 and disrupting fatty acid β-oxidation, thereby promoting liver fat accumulation. SIRT1 is known as a longevity gene, since it regulates the longevity effect induced by caloric restriction, and it enhances DNA-base-excision repair and produces unique metabolites (Rahman and Islam, 2011). To investigate whether and how 40-Hz light flicker may improve alcoholic fatty liver degeneration *in vivo*, we employed a mouse model of acute alcoholic steatosis *via* triple intragastric administration of ethanol to the stomach. We investigated the effects of 40-Hz light flicker on SIRT1 and p-AMPKα. Our data demonstrated that 40-Hz light flicker remarkably increased the activities of SIRT1 and p-AMPKα. AMPK regulates the balance of lipid metabolism, and the p-AMPK activation increases lipid β-oxidation (Woods et al., 2017). SIRT1 deacetylates and activates the upstream kinase of AMPK, LKB1 (Yao et al., 2017). LKB1 activates AMPK through phosphorylation. In addition, activated AMPK can activate nicotinamide phosphoribosyl transferase through transcription, which in turn activates SIRT1. SIRT1 regulates lipid homeostasis and mediates fatty acid-β oxidation through interaction with peroxisome proliferator-activated receptor alpha (PPARα) (Sharples et al., 2015). The expression of PPARα positively correlates with the expression of SIRT1 (Avila et al., 2016). In alcohol-exposed mice, we found that 40-Hz light flicker enhanced liver SIRT1 expression; therefore, we speculate that 40-Hz light flicker also increased PPARα expression. Ethanol inhibits SIRT1, which leads to inhibition of AMPKα phosphorylation. However, AMPKα also regulates SREBP1 activity, which controls lipid synthesis. In our present study, 40-Hz light flicker suppressed SREBP1 expression and stimulated p-AMPKα in alcohol-induced steatotic livers, suggesting that 40-Hz light flicker modulated lipid metabolism by inhibiting lipid biosynthesis and promoting lipid β-oxidation.

Several processes play a role in coordinating the expression of circadian-associated genes—such as those involved in fatty acid synthesis/oxidation, glucose metabolism, mitochondrial function, and tricarboxylic acid cycle (Panda et al., 2002)—which precipitate diurnal variations in many metabolites (Eckel-Mahan et al., 2012). There are many key transcriptional regulators of lipid metabolism. These transcriptional regulators are present in both adipose and liver tissues and are controlled by the circadian clock. Peripheral circadian rhythms are critical for maintaining organ physiology and function. Research has shown that circadian rhythm disorders can lead to lipid metabolism disorders, obesity, as well as metabolic diseases. It has been shown that a high-fat diet significantly inhibits the hepatic circadian clock, even before the development of obesity (Kohsaka et al., 2007). Additionally, acute alcohol exposure disrupts the molecular clock of the liver. Activated SIRT1 is directly phosphorylated by AMPK kinase-associated cryptochrome 1 and is involved in the regulation of lipid biosynthesis. Therefore, we were intrigued whether 40-Hz light flicker regulated alcoholic liver steatosis *via* the circadian clock. As expected, ethanol decreased BMAL1 and CLOCK protein levels, which were suppressed by 40-Hz light-flicker administration and was accompanied by PER2 degradation in the liver, suggesting that 40-Hz light flicker may correct circadian gene disruption in alcoholic liver injury. SIRT1 triggers high transcription of some clock-related genes, thereby participating in the control of circadian rhythms. SIRT1 expression exhibits circadian oscillations (Asher et al., 2008) (Nakahata et al., 2008) (Nakahata et al., 2009). In this study, we focused on the inhibition of alcoholic liver injury and its possible molecular mechanisms. In particular, it is possible that 40-Hz light flicker modulates alcoholic liver injury *via* activation of SIRT1 and BMAL1. As expected, 40-Hz light flicker increased the expression levels of circadian-related proteins in the SCN. Interestingly, alcohol did not influence Per2 mRNA in the SCN as it did in the liver, indicating that further studies are needed to determine the effect of 40-Hz light flicker on PER2 protein in the SCN.

The discovery of mechanisms for protecting against ethanol-induced liver injury is an important step towards the development of effective treatments for liver fibrosis, before overt clinical symptoms develop. It was previously reported that stimulation of 40-Hz neural activity *via* noninvasive administration of 40-Hz light flicker triggered the transformation of microglia into an engulfing state and inhibited Aβ, the latter of which contributes to neurotoxicity in Alzheimer's disease (Singer et al., 2018).

In this study, 40-Hz light flicker prevented alcoholic hepatosteatosis through activating SIRT1 and resetting the circadian clock. To the best of our knowledge, this is the first report to provide evidence that 40-Hz light flicker can reprogram the circadian clock to improve alcohol-induced liver injury. RNA-seq analysis indicated significant changes in expression levels of genes related to AMPK signaling. Therefore, we speculate that 40-Hz light flicker exerts its protective effects on ethanol-induced liver injury by activating SIRT1.

In conclusion, our study demonstrates that 40-Hz light flicker modulated central circadian control in the brain by modulating the activity of SIRT1 (**Figure S2**). This effect suppressed alcohol-induced lipid accumulation in mice liver. Furthermore, 40-Hz light flicker decreased SREBP1 activation following alcohol administration, indicating that 40-Hz light flicker may be an effective therapy for alcoholic liver steatosis.

## Data Availability Statement

The raw data supporting the conclusions of this manuscript will be made available by the authors, without undue reservation, to any qualified researcher.

## Ethics Statement

The animal study was reviewed and approved by the Institutional Animal Care and Use Committee of Shenzhen University.

## Author Contribution

YZ and YYa are the primary investigators in this study. RM, WZ, and QD participated in part of animal experiments. YYi, AZ and SZ participated in part of data analysis. JM and YZ designed this study and wrote the manuscript as corresponding authors. JM prepared all the light irradiation devices needed for the experiment.

## Conflict of Interest

The authors declare that the research was conducted in the absence of any commercial or financial relationships that could be construed as a potential conflict of interest.

## Supplementary Material

Figure S1 Schematic of the experimental setting. (A) The following parameters of light were used during the experiment: central wavelength of 462.6 nm and a color temperature of Tc ≥ 25,000 K. (B) Diagram of sample stimulus conditions (40 Hz) and intensity of light (3 mW/cm^2^). (C) Animal experimental procedures.

Figure S2 A schematic representation showing the partial prevention of ethanol-induced liver lipid deposition and inflammation *via* 40-Hz light flicker.

Figure S3 Effect of 40-Hz light flicker on Sirt1 mRNA. The mRNA levels of Sirt1. ^#^*P* < 0.05 significantly different from control group; ^*^*P* < 0.005, ^**^*P* < 0.01, significantly different from ethanol-alone group; one-way ANOVA followed by Tukey's test. All of the histograms represent the mean ± SD of five independent assays.

## Abbreviations

ALD, alcoholic liver disease; ALT, alanine aminotransferase; AMPK, AMP-activated protein kinase; BMAL1, brain and muscle arnt-like protein-1; CLOCK, circadian locomotor output cycles kaput; PER, period; SCN, suprachiasmatic nuclei; SIRT1, Sirtuin 1; SREBP1, sterol regulatory element binding protein-1; TG, triglycerides.
