# Supplementary figures and images for: Noninvasive 40-Hz Light Flicker Rescues Circadian Behavior and Abnormal Lipid Metabolism Induced by Acute Ethanol Exposure via Improving SIRT1 and the Circadian Clock in the Liver-Brain Axis
Source: Front Pharmacol. 2020 Mar 25;11:355. doi: 10.3389/fphar.2020.00355 (PMC7109315; doi:10.3389/fphar.2020.00355)

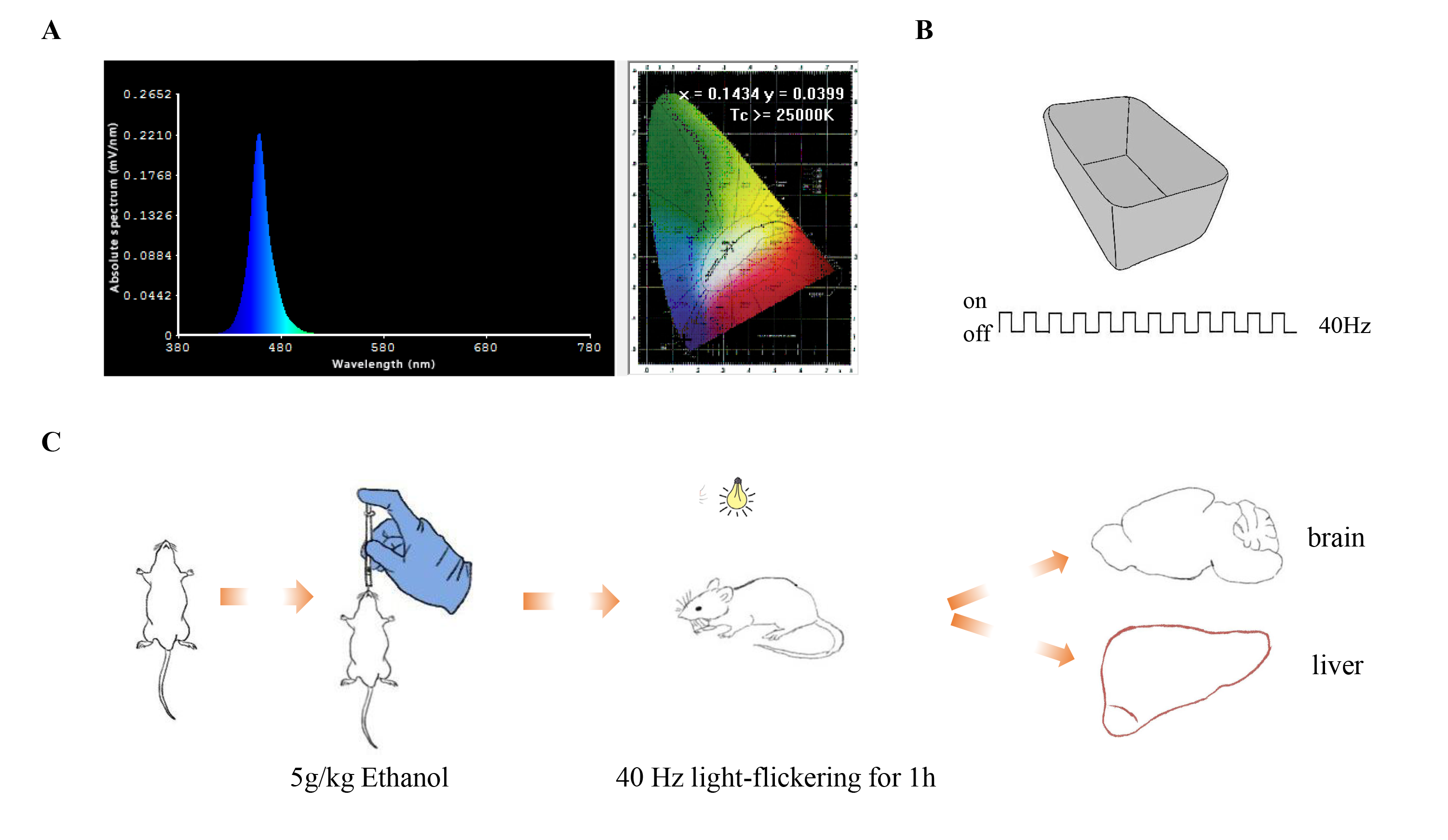

Supplement: Figure S1 — Schematic of the experimental setting. (A) The following parameters of light were used during the experiment: central wavelength of 462.6 nm and a color temperature of Tc ≥ 25,000 K. (B) Diagram of sample stimulus conditions (40 Hz) and intensity of light (3 mW/cm2). (C) Animal experimental procedures. [file Image_1.tif]

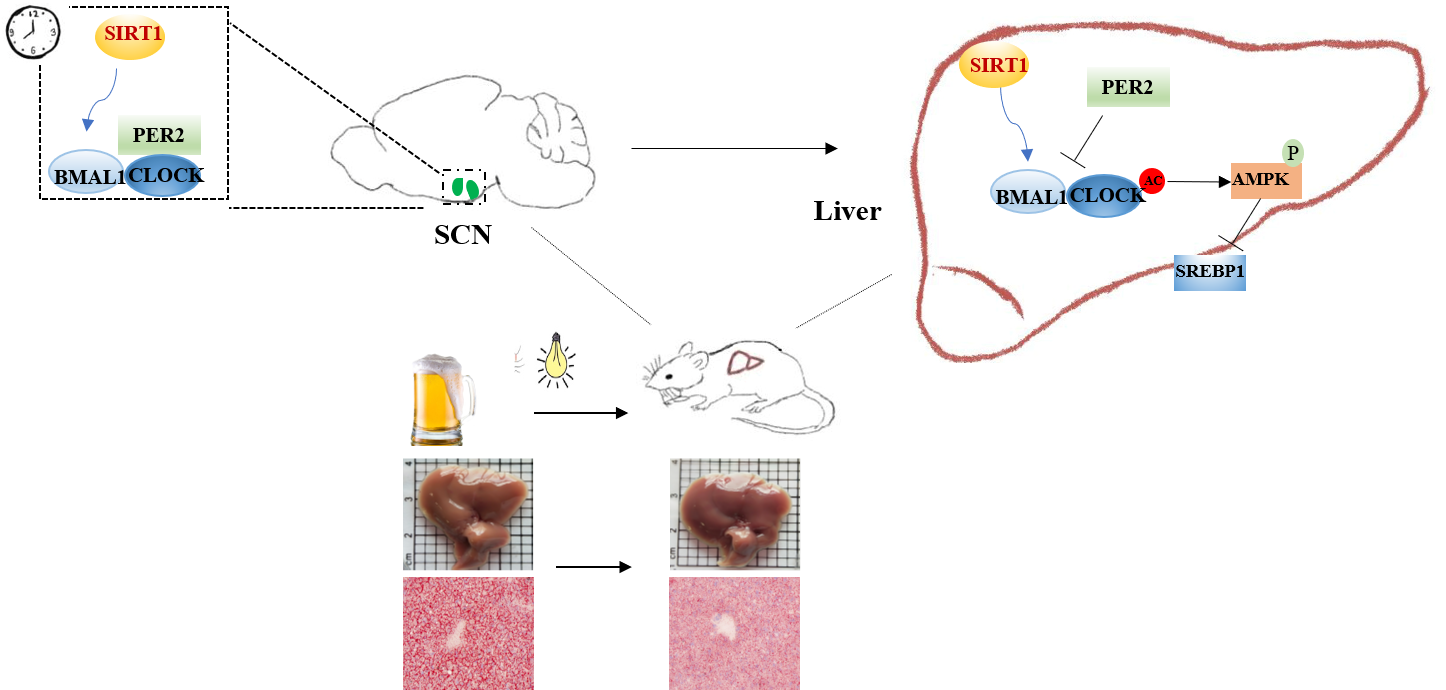

Supplement: Figure S2 — A schematic representation showing the partial prevention of ethanol-induced liver lipid deposition and inflammation via 40-Hz light flicker. [file Image_2.tif]

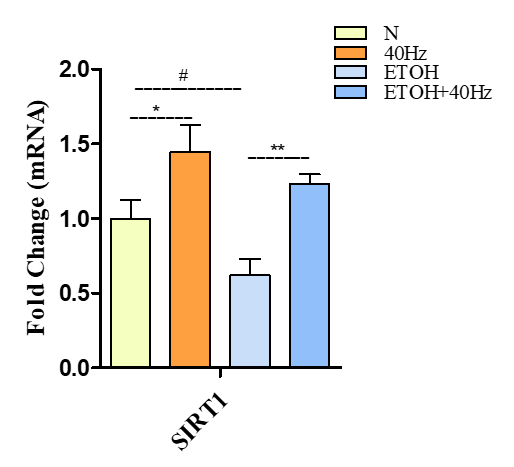

Supplement: Figure S3 — Effect of 40-Hz light flicker on Sirt1 mRNA. The mRNA levels of Sirt1. #P < 0.05 significantly different from control group; *P < 0.005, **P < 0.01, significantly different from ethanol-alone group; one-way ANOVA followed by Tukey's test. All of the histograms represent the mean ± SD of five independent assays. [file Image_3.tif]
